# What do we owe feral animals?

**DOI:** 10.1017/awf.2026.10119

**Published:** 2026-10-07

**Authors:** Christian Gamborg, Clare Palmer, Janne Winther Christensen, Alistair Lawrence, Peter Sandøe

**Affiliations:** 1 Department of Food and Resource Economics, Faculty of Science, University of Copenhagen, Denmark; 2 Department of Philosophy, Texas A&M University, College Station, USA; 3 Department of Animal and Veterinary Sciences, Aarhus Universitet, Denmark; 4 Roslin Institute, The University of Edinburgh Royal Dick School of Veterinary Studies, UK; 5 Department of Veterinary and Animal Sciences, Faculty of Health, University of Copenhagen, Denmark

**Keywords:** Animal welfare, domestic animals, ethics, feral animals, Sable Island, Hirta, wild animals

## Abstract

A common ethical view, widely reflected in legislation, accepts duties of care for domesticated animals under human control, but does not extend such duties to free-living wild animals. This paper addresses the question of what is owed to a group of animals that falls between these two groups, i.e. *feral* animals that have domesticated origins, but that now live largely independently. We discuss the question in light of two contentious cases: feral horses on Sable Island in Canada and Soay sheep on the island of Hirta in the Scottish Hebrides. Both populations are located on resource-restricted islands with periodic severe challenges to food supply. These challenges have led to significant ethical disagreement about whether to treat the animals as wild and leave them alone, or to accept duties of care by providing supplementary food or controlling population size to reduce suffering. For the Soay sheep, and to some extent for the Sable Island horses, the debate seems to centre around whether they are perceived to be wild or domestic animals. We argue that management of feral animals should consider more than the assigned domestic or wild ‘status’ of the animals and seek to engage stakeholders in making decisions based on multiple and, to some degree, competing values, including animal welfare, wildness, biodiversity, and other conservation values.

## Introduction

Animals are often divided into two categories, ‘domestic’ and ‘wild’, depending on their relationship with humans. However, how to understand domestication and what makes an animal ‘domestic’ is highly contested. We will use the term in the following way: Domestic animals are (roughly) characterised by two conditions. Firstly they are domesticated, i.e. are genetically adapted to living with humans through a process of evolution in response to a human niche (Lord *et al.* 2025) or direct selection for tameness and/or use by humans and, secondly, they live under various forms of human control and care (for instance, as companion and production animals). Wild animals, on the other hand, are not domesticated, and largely live independently of the comprehensive human control and care that characterise domestic animals (although, as will be discussed below, they may still be subject to more limited or intermittent forms of control, such as pest management or hunting regulation, without thereby ceasing to be wild). This distinction is more than just conceptual; it also underpins legal frameworks and ethical norms for how animals should be treated. In many countries, the law requires provision, and in particular the provision of food and shelter, for domesticated animals in human care (Carmichael 2024; Miranda-de La Lama & Estévez-Moreno 2025). But provision is only required for wild animals when they are in zoos or other forms of captivity. Widespread ethical norms are roughly in line with such legal requirements; while neglecting to feed a pet dog is widely regarded as morally wrong, no such concerns are normally expressed about not feeding wild urban foxes, even if the foxes lack access to food. It may even be widely considered as problematic to feed wild animals living close to humans when these are perceived as pests, or where feeding changes wild animal behaviour. Of course, these legal and ethical distinctions can be challenged, as we will discuss later (e.g. Horta & Terance 2023). In particular, people appear willing to support some interventions in the lives of wild animals — especially individual-level assistance — though support drops sharply for large-scale or ecosystem-altering interventions (Sleegers *et al.* 2025; Campbell-Ward *et al.* 2026). Thus, some wild animals who live close to humans, for example wild birds in temperate or cold areas, are widely fed by humans during winter, based in part on concern for their welfare (Cox & Gaston 2016). For most wild animals, however, population dynamics involving regular seasonal starvation and mass mortality is commonly seen as normal and not requiring intervention.

While this understanding of a domestic/wild divide has many uses, it is also problematic, both conceptually and in terms of its alleged ethical implications. In this paper, we focus on one particular problem: that the domestic/wild dichotomy does not have an obvious place for animals who fall between these categories. And since, as we have pointed out, the domestic/wild divide also often anchors legal and ethical frameworks, this means that there is scant guidance about how these ‘in-between’ animals should be treated in a legal context. We will focus on *feral* animals: animals that were formerly domesticated, but who now live outside human care and breeding control. Our main goal is to answer the question: What is owed to such feral animals in ethical terms? We argue that a more complicated view than a simple divide between ‘wild’ and ‘domestic’ animals is needed to inform decisions about what is owed to feral animals. But this does not mean that we reject the relevance of ‘wild’ and ‘domesticated’ altogether, unlike those animal ethicists who argue that only the intrinsic capacities and related interests of animals matter ethically; we will return to these views later.

We will first look at several existing possible frameworks for categorising animals, and draw from these to think about feral animals. Then we will consider two cases that give or have given rise to controversy: the feral horses (*Equus ferus caballus*) of Sable Island in Canada, who have been on the island since the late 1700s (Plante *et al.* 2007); and the feral sheep (*Ovis aries*) population on Hirta, St Kilda’s main island in the Scottish Hebrides, that has lived in the Hebrides with little or no human management for thousands of years (Fleming 2021). These animal populations have somewhat different histories — the horses, though longtime residents, are more recent arrivals on their island — but both are descendants of domestic animals, and thus have domestic origins, and both are located on islands that lack natural predators, thus periodically facing severe challenges in terms of food supply. These resource challenges have generated significant ethical disagreement, particularly in the case of the sheep, about whether the animals should be left alone, like wild animals, or whether there is a legal and an ethical responsibility to provide supplementary food, and/or to control population size, in order to prevent suffering. These disagreements — in part — depend broadly speaking on whether these populations are understood to be ‘wild’ or ‘domestic’.

Our focus is ethical (a separate discussion could be had concerning how relevant welfare legislation should be applied to these animals; however, we will not develop this aspect here) but we do not aim to provide ‘instructions’ about what to do in these cases. Still, we argue that a more complicated view than a simple divide between ‘wild’ and ‘domestic’ animals is needed to inform decisions about what is owed to feral animals. We argue that context is ethically relevant, not just equal consideration of similar interests (a position which has been proposed by some scholars within animal ethics, as we will discuss later). Rather, we defend a more contextual and pluralist approach that considers animal suffering, animal agency — and other aspects of animal welfare — as well as other social and ecological values. Since disagreement among different stakeholders (including veterinarians, conservation and wildlife biologists and animal welfare groups) makes the management of feral animals controversial, we hope that this paper will make a useful contribution to discussions about the management of these animals.

## Categorising animals

There have been several previous attempts to categorise animals in a more complex way than a simple wild/domestic binary. Swart (2021), for instance, proposed a framework where animals are categorised according to, respectively, their degree of dependence and adaptation to the human system. This yielded four categories of animals: high dependence/high adaptation (e.g. animals kept by humans, for instance farm animals); high dependence/low adaptation (e.g. threatened animals, such as polar bears when sea ice melts); low dependence/high adaptation (e.g. recolonising animals, like wolves in Europe) and low dependence/low adaptation (e.g. free living wild animals). However, feral animals do not fit neatly into any of these four categories. So, while there are contexts where this framework might be useful, it is not very helpful for the more specific question being addressed here. Another categorisation, proposed by Donaldson and Kymlicka (2011), divides animals into three groups: domesticated; wild; and liminal. The liminal category primarily refers to undomesticated animals who share living space with people, such as urban wildlife. This three-fold categorisation again is useful, but does not specifically consider feral animals who occupy an underspecified position for Donaldson and Kymlicka; they are “*in the expanding liminal zone where the boundary between wild and domestic is blurred*” (Donaldson & Kymlicka 2016). Another framework, by Palmer *et al.* (2024), operates with four categories, separating out animals’ domestication status from their dependence/independence on humans. We present a modified version of this categorisation in Table 1.

**Table 1.** Categorising animals according to domestication status and reliance on humans (modified from Palmer *et al.* 2024) Table 1. long description.

	**Dependent on humans** (managed by humans)	**Independent of humans** (no human care)
**Domesticated** (genetically adapted through breeding and in other ways to living with humans)	**Domestic animals** (e.g. companion dogs and cats, farmed sheep, cattle, pigs)	**Feral animals** (e.g. feral cats, mustangs, feral goats) **Rewilded animals** (e.g. hardy Exmoor ponies in rewilding projects, Heck cattle, Konik ponies, iron age pigs)
**Non-domesticated** (no significant genetic adaptation to living with humans and no deliberate selective breeding^1^)	**Zoo and captive wild animals** (e.g. caged big cats, elephants in sanctuaries)	**Wild animals** (e.g. black bears, wolves, wild cats, mustelids, bats)

1 As noted in Lord *et al.* (2025), animals such as house sparrows can become genetically adapted to living close to humans. Such effects have also been documented for urban wildlife (Zsolnai *et al.* 2022).

This table picks out two categories of animals that are not clearly ‘wild’ or ‘domestic’ — those who are not selectively bred or genetically adapted to live alongside humans, but who nonetheless live under human care (i.e. zoo and captive wild animals); and those who have been directly or indirectly selected for use, at least at some point in the past, but who now live largely independent of human care in terms of feeding and housing, i.e. feral animals and those used in rewilding projects where they are not provided care but are treated as wild animals despite their domesticated origins. The difference between feral and rewilded animals is that in the case of rewilded animals the change is deliberate and is taking place within a specific nature conservation context (Gamborg *et al.* 2010; Jepson 2025). For the rest of this paper we will only focus on feral animals.

This categorisation highlights important ways in which feral animals have been shaped by, but are also independent of, humans. One way in which such categorisations matter, as we will go on to suggest, is that at least some of these factors are relevant to what might be owed to feral animals *ethically.* Before we turn to this ethical question, we will first look more closely at how feral animals have been defined.

## Our focus: Feral animal populations

Feral animals are descendants of domestic animals who live in the ‘wild’ and reproduce without direct and/or intentional human oversight, control or support (MacDonald *et al.* 2017). Typically, these animals of domestic origin display wild-type *behaviour* towards humans — in the sense of not being *tame* (Daniels & Bekoff 1989). They may also be described as ‘wild’ in the sense of being free-roaming or ranging. (Some scientific disciplines have more specialised ways of referring to members of species as feral — for instance, small animal veterinarians may mean by feral (companion) animals those that are unsocialised to humans, and this designation might change over time if the animal later accepts human contact). Sometimes these animals change status back to being domestic; for example, feral cats or horses may be tamed, live with and be cared for by humans and their offspring will end up as ordinary domestic animals.

Sometimes feral animals exist as separate *populations* where genetic and morphological changes happen over time. Here, feralisation is typically seen as the reverse process to domestication — where domestication is understood as a process, involving indirect or direct genetic selection, and human care and control (Purugganan 2022). It should also be noted that in some places, most notably Australia, the term ‘feral’ can also be used to denote populations of *wild* animals, such as cane toads and red foxes, which were introduced to the continent, and who have had negative impacts on native flora and fauna (McEwen 2007). We will not use the term feral in this last sense here, but note an extensive literature on feral animals so understood (e.g. Hampton & Hyndman 2019). The Australian context, though, does raise the point that in many places, feral animals (understood in all ways) are characterised as ‘invasive’ or as ‘pests’ because of their non-native origin and their adverse impacts on either native wildlife, biodiversity or livestock.

## Two cases

Clearly, there are numerous types of feral animals. Here, we will focus specifically on *populations* of feral animals, through the lens of two cases of ungulate herbivores on islands without predators and with limited resources: the Sable horses and the Soay sheep. When considering feral animals at population level, we are looking at processes spanning at least one generation and usually many more. These populations have sparked considerable controversy about whether, as non-native animals, they belong on the islands at all; and whether, as feral animals, they are best understood as ‘wild’ or ‘domestic’ in terms of the kinds of care or management they should receive. Moreover, there are important differences in the two cases between the discussions that have taken place relating to the management of the animals.

### The Sable horses

Sable Island is a small (36 km long, 2 km wide at its widest) remote, crescent-shaped sand-bar in the North Atlantic Ocean off the coast of Nova Scotia, Canada. Located in the middle of a major shipping route, it was once famed for shipwrecks. In 1760, horses and other species (including hogs and goats) were brought to the islands, with the goal that the animal populations would grow and could periodically be taken off the island and sold (Frasier *et al.* 2016). However, only the horses survived, becoming a well-established free-ranging population not maintained as domestic livestock in any conventional sense but with periodic human interactions, e.g. removal of individuals for food or labour (Welsh 1975). Later, during the first half of the 19^th^ Century, other horses, mostly stallions, were brought onto the island with the hope of breeding higher value horses for “harvesting” (Cabrera *et al.* 2017), but there was never a clear transition from a managed to an unmanaged population (P McLoughlin, personal communication 2026).

By the 1950s, ecologists expressed concern that the growing horse population was damaging the island, proposing plans to export and cull them all (Freedman 2015). Public pressure, including a letter-writing campaign by children, led to their protection by law in 1961 as part of the Canadian Shipping Act (Plante *et al.* 2007). This law, which formalised a strict non-interventionist approach, meant that horses could not be rounded up and sold off the island, nor could new horses be brought in for breeding purposes. And as a “naturalised population” neither could the horses be fed, touched, or offered veterinary care. This distinguishes the Sable Island population from most other free-ranging horse populations where some degree of care or population control can be provided (Kirkpatrick & Turner 2007). In 2013, Sable Island was designated a national park reserve, and management moved under the Canada *National Parks Act*, recognising the horses as part of the island’s naturalised ecosystem and managed as wildlife, keeping the strict non-intervention policy.

Several factors make this population unusual among feral horses; first it has had much longer (250 years) to adapt genetically than most feral horse populations; secondly, it has now been completely unmanaged for well over 60 years (Simpson 2015) and thirdly, it is a ‘closed’ population, in that no new horses from outside have recently been added. Indeed, over time, the horses have genetically diverged from their original breeds such that they now form a population itself regarded by some as being part of the heritage value of the island (Villeneuve 1997), and seen to have conservation value in itself (Plante *et al.* 2007). Consequently, the population exists without predation, human interference, and guild competition, i.e. others exploiting the same class of environmental resources in similar ways (McLoughlin *et al.* 2016).

**Figure 1.**
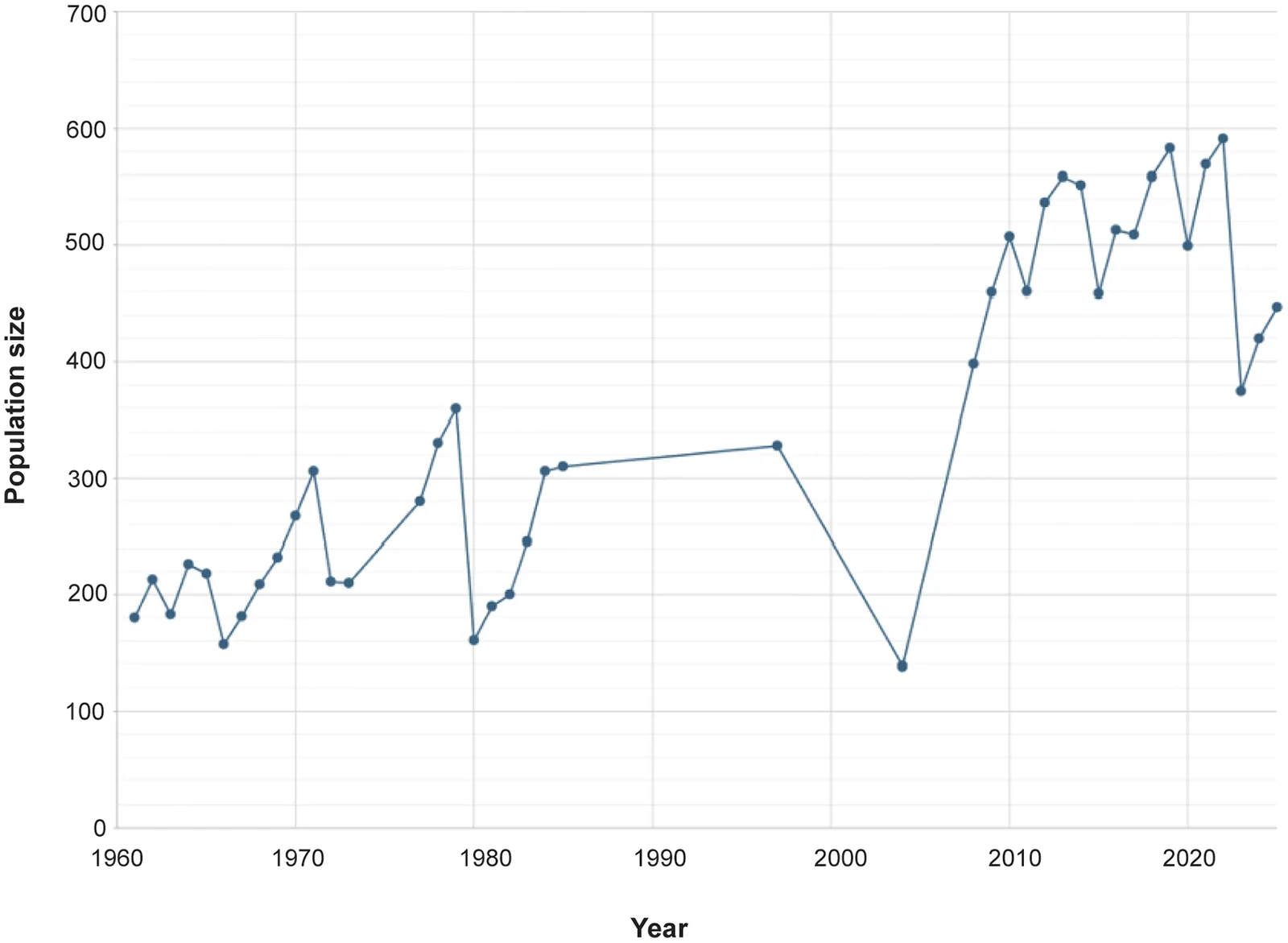
Variation in population size of Sable Island horses (1961–2025). Data originate from air photographs or ground-census counts (Frasier *et al.* 2016; P McLoughin, personal communication 2026). Figure 1 long description.

The initial size of the horse population, when introduced in 1760, was approximately 60. Over time, after 50+ generations, the population has grown, although not in any steady way as there have been years with very significant population drops and others with significant rises, reaching a peak population of close to 600 in 2022 (Figure 1). In 2024, the estimated number of horses was 420. Recent studies found that in the summer, when resources are plentiful, most horses are in “moderate to excellent body condition” (Jenkins *et al.* 2020). Part of the increase in population size may be explained by a change in vegetation leading to improvement in forage quality over time (McLoughlin *et al.* 2016). Mortality is largely seasonal, with poor weather and starvation from lack of resources in winter (which mostly affects foals and yearlings), combined with high parasite loads. The average winter mortality rate is around 12%, but the rate varies with weather conditions; for example, in 2023, the mortality rate was more than double the average, as can be seen from the fluctuations in population size (Figure 1). In comparison, a well-studied group of wild horses, the Pryor Mountain wild horses in Montana in the US, showed mortality rates of on average approximately 12% but in bad winters up to 18%, and in one extreme winter as high as 51% (Roelle *et al.* 2010).

Older horses may also die of starvation when their teeth are ground down due to the tough marram grass and exposure to sand over time, or from colic caused by ingesting sand. The horses also, as mentioned, carry high parasite loads, including parasites no longer found in North American domestic horses — although they also are free from parasites now common in domestic horses. These parasites may affect welfare, but they do not seem (alone, at least) to be a major cause of mortality (Jenkins *et al.* 2020). There are also “modest levels” of inbreeding depression — that is, loss of fitness due to the lack of genetic diversity from being a closed population (Colpitts *et al.* 2024). All this explains why the mean life expectancy of the horses on Sable Island is short, at an average of 6 years for males and 4.5 years for females (https://hakaimagazine.com/features/romance-politics-and-ecological-damage-the-saga-of-sable-islands-wild-horses/). In comparison, the mean life expectancy of feral Assateague horses is approximately 10 years (Kirkpatrick & Turner 2007) and even higher in feral Konik horses in Poland (19.6 years for stallions and 17.8 years for mares; Górecka-Bruzda *et al.* 2020).

As already said, management policy for Sable Island horses essentially treats them more like wild than domestic populations. Indeed, not only is helping them not *required*, as is usual for wild populations, it is not *permitted.* However, unlike many wild populations, regulating them (for instance by hunting) is *also* not permitted; even though they have no natural predators on the island. They are subjects of multiple non-invasive scientific studies that measure factors such as mortality, social structures, resource use and parasite load (Frasier *et al.* 2016). It is clear that the horses are protected from being removed for sale or hunted due to past public concerns about killing them. However, official resources do not explain why they are not to be helped, and why humans are required to keep their distance. Presumably, this is based on valuing the horses’ “natural lives” apart from humans and that they are a “symbol of wildness” as suggested by a journalist from the *Haikai* magazine (https://hakaimagazine.com/features/romance-politics-and-ecological-damage-the-saga-of-sable-islands-wild-horses/).

Nonetheless, their management may lead to controversy, in two distinct directions. One concern is that the horses are not “natural” to the island; and that, as a non-native species, they destroy the island’s native biodiversity — though in fact there are few studies actually indicating significant effects (Freedman *et al.* 2011). Since Parks Canada has a remit to remove “invasive alien species”, this, it has been argued, should apply to the horses too (for instance, in a news article from the Canadia Broadcasting Company in 2014: https://www.cbc.ca/news/canada/nova-scotia/sable-island-should-the-feral-horses-be-removed-1.2756920).

The other concern, which to the knowledge of the authors of this paper has not so far given rise to controversy, is the welfare of the horses themselves. While they can mostly live well in the summer, high mortality in winters, mainly affecting foals, yearlings and older horses, suggest that these horses undergo considerable suffering prior to death. It is, for example, a reasonable assumption that starvation causes suffering (Leyton 1946; Harvey *et al.* 2023). More generally, the short average lives of Sable Island horses, compared to other feral horse populations, suggests suffering faced by many of the horses. This kind of suffering would not be legally acceptable or ethically tolerated for domestic horses. On the other hand the horses are free to carry out species-specific horse behaviour without restriction, including mating, parenting, social relationships, foraging and freedom of movement — unlike most domestic horses. This suggests that the horses likely experience good welfare in terms of meeting their behavioural needs during parts of the year, but may also suffer at times due to lack of food, water and shelter, and due to untreated painful conditions, such as injuries and sand colic.

These concerns illustrate the kind of management difficulty we outlined at the beginning of this paper. These horses are neither clearly wild nor domestic. They are in a context selected by people: a resource-restricted, predator-free island, but they could be described as functionally wild. This creates welfare concerns for the horses, and, perhaps, biodiversity costs.

### Soay sheep

**Figure 2.**
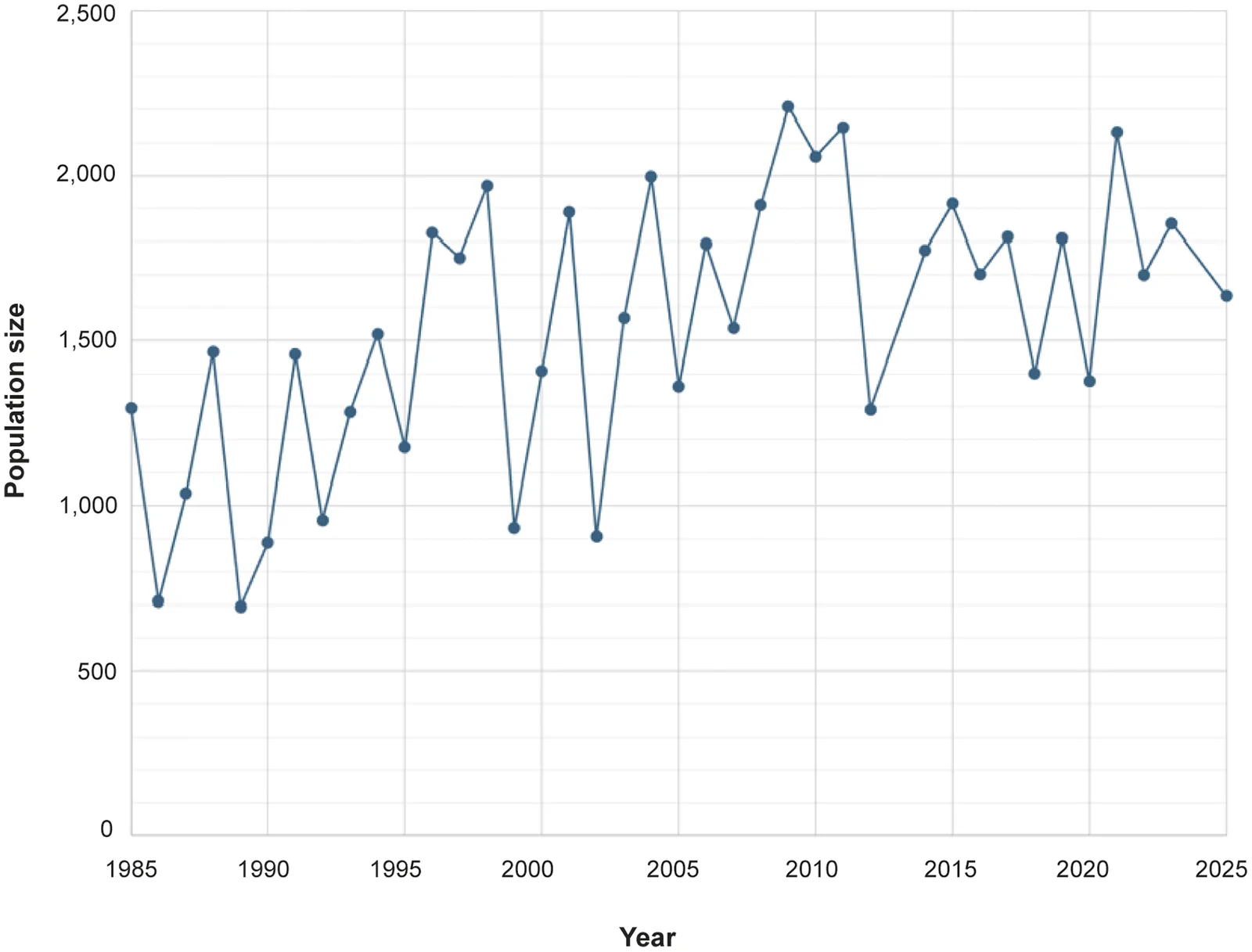
Variation in population size of Soay sheep on Hirta Island; data originate from annual census (D Nussey, St Kilda Soay sheep project, personal communication 2026). Figure 2 long description.

Sheep (*Ovis aries*) were among the first herded livestock species domesticated in southwest Asia about 8,000–10,000 years ago (Vigne 2011). The population at issue in this case descends from sheep brought by early settlers to islands off the west coast of Scotland. These sheep are thought to have lived independently for 2,000–3,000 years as a feral population on the uninhabited island of Soay in the St Kilda archipelago (80 miles west of the Scottish mainland). Residents from the nearby island of Hirta, an adjacent island of about 670 hectares, would cross to Soay to harvest the sheep for wool or meat. In the 1930s, however, when Hirta was abandoned by people (who took their livestock with them), some of the sheep were moved from Soay to Hirta where they have subsequently lived without human care. All sheep derive from Soay and constitute a primitive breed — related to the European mouflon — that is part of the cultural heritage of the islands, as documented in the UNESCO World Heritage Status citation of the islands (State of Conservation of World Heritage Properties in Europe undated). In 1952, the Soay sheep became the subject of an ongoing scientific population study, though not one that involved any form of management. The ongoing scientific study has found significant fluctuation in the Soay sheep population (Figure 2). Recently there are indications that the island is supporting a larger population (Crawley *et al.* 2021). In some years, such as 1998–1999 and 2011–2012, however, the population more than halved due to fluctuations in carrying capacity as the sheep died due to a complex combination of food limitation, exposure to winter weather and parasites, among other things (Pilkington *et al.* 2024).

These population fluctuations have recently become controversial. A briefing from 2021 for the Citizen Participation and Public Petitions Committee on Petition PE2021, lodged by two local veterinarians from Uist in the Outer Hebrides (islands between the Scottish mainland and the St Kilda archipelago), argued that the Scottish government, and the National Trust for Scotland (NTS) that owns the islands, have a duty of care to the Soay sheep. These animals, they argue, are “unsuited to life unmanaged” and “domestication has left sheep reliant on man for population control” (Buckland & Charlesworth 2021; p 3). They base their argument on the Welfare of Farmed Animals (Scotland) Regulations (2010; p 22–27) which states that “animals must be fed a wholesome appropriate diet which is appropriate to their age and species, and which is fed to them in sufficient quantity to maintain them in good health, to satisfy their nutritional needs, and to promote a positive sense of wellbeing”. Since the Soay sheep, according to the two veterinarians, are domestic (farmed) animals, this regulation, they argue, should apply to them.

In contrast, the National Trust for Scotland and those running the current scientific research project on the Soay sheep from Edinburgh University argued that the sheep have been living virtually unmanaged for thousands of years, and are not closely related to modern domesticated breeds; they should be seen instead effectively as a wild population (Buckland & Charlesworth 2021). Since wild mammal species, such as deer, can also have high die-off rates in the winter, this is a pattern that should be expected of wild sheep too.

The Scottish Government deemed that “protected” animals (those for whom there is a duty of care and provision) include “the kinds of animals whose collective behaviour, lifecycle, or physiology has been altered as a result of their breeding and living conditions being under human control for multiple generations” (Buckland & Charlesworth 2021; p 7). The Government also maintained that this generally applies to feral members of those domesticated species too — such as “feral cats, sheep, goats or ponies”. This is so because “when man has made an animal dependent on him, then the animal should continue to be protected” (Buckland & Charlesworth 2021; p 7). However, this judgment, it was argued, does not apply to Soay sheep, who are an “exception” to this consideration. While accepting that they were “originally kept as domesticated animals” because their “ancestors have adapted to life on St Kilda over many generations” the Soay sheep are “not dependent on humans in the same way that more recently escaped or released domesticated animals would be” (Buckland & Charlesworth 2021; p 7).

The sheep are seen as an integral part of the St Kilda Archipelago,which was initially inscribed on the World Heritage List in 1986 for its outstanding natural features and wildlife, and expanded in 2004 to also cover cultural value, and hence the sheep. The sheep are characterised by UNESCO as being one of “two isolated populations of one of the most primitive domesticated sheep in existence, living in a feral condition completely unmanaged by man” (State of Conservation of World Heritage Properties in Europe undated). So, the sheep in terms of being “primitive domesticated”, “in feral conditions” and “completely unmanaged” should, according to the Scottish Government, *not* be treated like other feral animals, but instead as if they were a wild population.

## Discussion of what is owed to the feral horses and sheep

What is owed to animals seems to depend on two things, what matters and what can be done, i.e. possible ethical concerns and viable management options to deal with these concerns. Multiple values are at stake here, as we will show below; these do not line up straightforwardly with whether the animals are ‘domestic’ or ‘wild’ but relate to a range of concerns about the lives and welfare of the animals, their relationships to humans, and the environments in which they are located.

### Possible ethical concerns

First, and most obviously, there is likely to be significant *suffering* in both populations. Starving to death, which many animals in both populations do, is surely the source of significant negative subjective experience (Leyton 1946; Harvey *et al.* 2023). On some ethical views, that alone is a matter of ethical concern.

Second, these two populations of animals, on relatively small islands with harsh winters, in the absence of predation and with limited availability of food, are bound to face events of seasonal starvation. But the reason the animals are in such circumstances is because humans put them there. So, there is some kind of *human causal responsibility* — though, it should be added, in both cases this is distant in time, and not the immediate responsibility of any person or group now alive. Nonetheless, from some ethical perspectives that historical causal responsibility has a degree of ethical relevance.

Third — particularly notable in the case of the Sable horses — is an idea about the value of ‘naturalness’ or ‘wildness’. However, this can be construed in different ways. On one understanding, Sable Island with horses, as we have seen, is part of a vision of the island — the free-roaming horses are the ‘spirit of wildness’ and are valued in that context. On another understanding, the horses — and the sheep — can be seen as ‘natural’ in the sense that they have freedom to live their own lives, following natural behaviour patterns, e.g. of foraging, mating, care for young and social structure, in a way that is very rare for domestic animals. This kind of animal agency can also be seen as a valuable part of good welfare; but it is distinct from the horses’ contribution to the wildness of the island as a place.

Fourth — an issue that arose with the Sable Island horses — there are concerns about negative *environmental effects* of horses on the island’s valuable ecosystems and unique forms of life (this worry seems to be in tension, to at least some degree, with the idea of the horses as expressing the island’s naturalness). There are no similar concerns with the Soay sheep; a study from 2021 concluded that the botanical composition of the plant communities remained much as it was 85 years ago, and that there was no evidence of over-grazing (Crawley *et al.* 2021).

Fifth, the horses and the sheep are unusual enough genetically, because of the isolation of their populations, to be of *conservation value* as ‘heritage breeds’ of exceptional hardiness. This matters because if interventions were made to actively manage populations, there would likely be evolutionary changes that would affect this conservation value.

Finally, both populations are the object of a longitudinal research effort in the field of population biology which, in the case of the Soay sheep, has lasted since the 1950s and involves the handling and tagging of all individuals in one-third of the island. These studies, being carried out over extended periods, have significant *scientific value* just because they are investigating what happens in the rare cases of closed populations living with restricted resources. Any more interventionist policy would significantly affect, or end, these scientific studies and their associated scientific value.

Many different concerns therefore apply here. Some relate directly to the animals’ welfare; others to relationships with humans not straightforwardly captured by the division into ‘wild’ and ‘domestic’; yet others relate to the animals’ context and environment.

### Management options to deal with the ethical concerns

The most straightforward option is to maintain a hands-off policy when it comes to managing the populations by not assisting the animals. The contrasting choice would be to remove all the animals from the islands. Then there are a variety of possible interventions that could be made in between (individually or in combination). One possible intervention would involve supplementary feeding in hard winters and/or the provision of veterinary care, at least to deal with high parasite load. Another would involve a reduction in population size, either by periodic removal of some animals, the use of pharmaceutical or surgical fertility control to control population size; or lethal control through culling (though this could be operationalised in different ways and at different times). The most plausible kind of interventions would combine elements of care and population control, given that the care would likely, over time, contribute to increasing numbers.

The actual feasibility of these options will depend on context. For example, in the case of Soay sheep, moving the sheep to the mainland does not seem a viable option. The sheep are difficult to catch, especially in large numbers (Hinch 2017), and the cost of shipping them is high. Moving them would also bring significant welfare costs from attempted capture, disrupting social relationships, and transportation. Trying to turn animals not used to human handling and to confinement into normal farm animals would likely be deemed unacceptable in terms of animal welfare. Thus, any movement off the islands would have welfare implications for animals hefted to environments over generations and used to freedom of movement and behaviour. Reducing numbers, then, would be more plausibly carried out by pharmaceutical fertility control or by shooting some of the sheep on the island. These are not equivalent options from a welfare perspective and may generate disagreement (see e.g. Gamborg *et al.* 2020). Fertility control may bring some welfare benefits relative to lethal control; lethal control may have other welfare benefits (minimal handling) and when it comes to the ability of the animals to express their natural behaviour lethal control may be preferable, since a quick kill need not compromise an animal’s ability to behave naturally up to the point of death and will not affect normal behaviour related to mating (Littin *et al.* 2014; Beausoleil *et al.* 2016; de Ridder & Knight 2024). Which regime is preferable partly depends on the values being prioritised: a compassionate conservation approach, for instance, would tend to favour fertility control to avoid taking life, though how to weigh the different aspects of animal welfare, lives and expression of natural behaviour in cases like this remains a matter of ongoing debate.

These options may nevertheless prove widely controversial to the public and other stakeholders, especially in the case of shooting — not only because animals are killed, but because sheep on Hirta have historically been managed under a general policy of non-intervention. Indeed, publics have been shown to resist lethal control even where practitioners consider it justified (von Essen & Redmalm 2023). In the specific case of the Soay sheep, the live public dispute is in fact over whether any active intervention — culling, supplementary feeding, or veterinary care — should be permitted at all, against the current default of non-intervention. Some stakeholders, though, are campaigning for proactive management, which could include culling.

In the case of the Sable Island horses it may be relevant to consider that some other feral horse populations, such as the horses on Assateague Island, are managed through contraceptives to avoid overpopulation (Kirkpatrick & Turner 2007), which can reduce suffering due to lack of food. Decisions on whether to do this could be based on an overall welfare assessment, such as the ten-stage protocol suggested by Harvey *et al.* (2020). However, as mentioned above, this kind of management would violate current Canadian law.

Which option to pursue here will be shaped by which values are prioritised, as well as concerns related to the best use of scarce economic resources. Continuing as things are, with no intervention, prioritises values relating to animal agency, the conservation value of populations, and/or the scientific research. Removal or culling of all the animals, on the other hand, would remove the possibility for scientific research on these rare populations and their conservation value, and would give rise to the ethical and animal welfare concerns mentioned above. In the case of removal of Sable Island horses, this might allow the recovery of original ecosystems, assuming concerns about their ecological impacts are justified.

Our purpose here is not to advocate for a particular way of managing these populations, but to argue that multiple values are at stake, and that these do not automatically line up with whether the animals are either ‘wild’ or ‘domestic’. None of these options is ethically neutral: each reflects a different balance among the values set out above. Some relate directly to the animals’ welfare, including their agency and freedom to carry out natural behaviour. Others relate to the animals’ relationships with humans — such as causal responsibility and created dependence which more closely track traditional ideas of ‘domestic’ animals, controlled and cared for by people. Yet others relate to the animals’ context and environment. As we argue in the *Animal welfare implications*, it is this plurality of values — as opposed to a single dominant value such as suffering — that should guide decision-making.

## Animal welfare implications

The two cases can help in thinking through more general welfare-related duties to feral animals: and they also make clear that the labels of ‘wild’ and ‘domestic’ do not, in themselves, contribute much to the discussion. These terms are better seen as proxies for a group of underlying values or ethical concerns — for instance, created dependence in the case of ‘domestic’ animals and agency in the case of ‘wild’ animals.

The two cases we discussed here uncovered a number of relevant ethical factors for thinking about what is owed to feral animals when it comes to animal welfare, including human causal responsibility and animal dependence on humans (which seem to underpin some legal frameworks); relieving animal suffering; freedom for animals to live out their own lives in their own ways; animal wildness; and the loss from death or killing animals.

Our attempt to de-emphasise ‘wild’ and ‘domestic’ in an ethical context should be distinguished from another approach that has become increasingly popular in animal ethics. Here, ‘wild’ and ‘domestic’ are similarly de-emphasised, but not in favour of uncovering a plurality of underlying values. Rather, such arguments focus on just one of the values we mention above: animal suffering. It is then argued that suffering is bad wherever it occurs, that similar suffering should be regarded as being of similar importance and if there are ways in which suffering could be prevented or relieved without causing worse suffering elsewhere, then we should act to prevent or relieve it (Horta & Teran 2023). On this account, the distinction between wild, domestic and feral no longer matters: the goal is to relieve the most suffering possible, wherever it occurs.

If we looked at our case studies from this perspective, there clearly are suffering-focused interventions that could reduce or end starvation and pain, and reduce parasite load in these two populations. This would likely involve a combination of occasional supplementary feeding, veterinary care, and pharmaceutical fertility control to prevent the population exceeding its range, not to mention lethal control if it is assessed that an animal’s body condition is extremely poor in a way that cannot be reversed.

Because these islands are so remote this would not necessarily be straightforward or cheap, but the animals’ confinement to the islands, together with their relatively low levels of interaction with other sentient animals, would at least make calculations about reducing overall suffering relatively straightforward (since they would not need to involve, for instance, predator or scavenger relationships). Probably, all interventions are complicated from a welfare perspective. Although the suffering-minimising approach has been dominating the ethical discussion about what we owe to animals living outside human control, we maintain that this approach neglects *other* important relationships and values that are at stake. It regards the cause of suffering as irrelevant, thus rejecting the moral significance of questions about how human activities brought the animals to where they are; and the understanding of animal welfare at issue here has a narrow focus on suffering, rather than also including positive welfare aspects including animal agency (see e.g. Browning & Veit 2023).

This is unfortunate, as one of the striking features of the lives of the Sable Island horses and the Hirta Soay sheep in comparison with many domestic animals *is* their agency: the degree of control they have over their own lives and the ability to make their own choices; including where to roam (Palmer & Sandøe 2018); which is recognised as a critical aspect of welfare (Špinka 2019; Rault *et al.* 2025). Part of the sheeps’ agency is to heft and voluntarily constrain themselves to a home range which is an area smaller than the whole island. As we have already suggested, it is surely plausible that being able to perform species-typical behaviours, and overseeing one’s own life — where to move, what to eat, with whom to mate and so on, is significant to animals, and forms an important part of their welfare. What’s more, agency is an aspect of animal welfare where wild and feral animals generally do better than domestic ones, whose lives are largely dictated by their human owners. That said, clearly when a sheep is starving or a horse is dying of sand colic the animal can no longer be said to *have* agency in the sense of being able to make choices; so agency is limited by weather, broader environmental conditions and food supply. Despite this, it is important to put the possibility of a ‘bad death’ in the context of having had a good life.

Recognising the importance of agency for welfare has implications for the management of feral animals. We mentioned above the possibility of using pharmaceutical fertility control in the case of the Soay sheep or Sable horses. This *would* reduce suffering (through hunger) and early death (whether through starvation or culling). But it also changes animals’ mating and reproductive behaviours, and the structure of populations, which in turn changes the social options for animals significantly, so reducing their agency (Gamborg *et al.* 2020). This does not mean that fertility control should always be rejected, but it has implications for feral animals’ lives that go beyond suffering understood in a narrower sense of fear and pains.

This focus on freedom, though does not necessarily mean that feral animals should always be left to fend for themselves. Neither does it mean that suffering does not matter. Even in wild populations, hands-off policies to protect welfare may be applied in a selective manner. For example, in the absence of predators it has been argued that lethal control of hyperabundant populations of wild herbivores is a welfare-friendly form of management (Hampton *et al.* 2019).

We do not take the greater attention given to welfare in this paper to imply that it trumps other values at stake. Our approach is pluralist rather than hierarchical: no value identified here — welfare, wildness, conservation, or scientific value — takes automatic priority over the others, and which prove decisive will depend on the specific case. Animal welfare receives more sustained discussion here largely because the conjunction of intense suffering and human responsibility related to re-occurring food shortage is the value most directly at stake in the day-to-day condition of these animals, and the one most amenable to detailed empirical characterisation. And so, this reflects the nature of our case studies rather than a claim about its moral priority.

The management of feral animal populations leads to difficult ethical discussions. We have argued for a more complicated view than a simple divide between ‘wild’ and ‘domestic’ (or even a three-way ‘wild-feral-domestic’ animal spectrum). Rather, we have suggested a more contextual approach, where decisions are made in the light of multiple and sometimes competing values. Our two cases were chosen partly because they are relatively straightforward: closed populations with identifiable but historically distant human responsibility for their situation, unaffected by competition with other land uses. Most feral animals are not in this situation. Free-roaming dogs, such as community and stray dogs, one of the most widespread examples of feral animals, typically engage in ongoing relationships with humans, relationships that can differ in nature (Smith *et al.* 2019). Mainland feral horse populations also differ: in the western United States, herd management areas often overlap with livestock grazing allotments, adding an ongoing dimension of resource competition largely absent from Sable Island (Rutberg *et al.* 2022). Extending our framework to such cases is an important task for future work.

Nevertheless, in the case of many feral animal populations, there is some causal story, as in the two cases here, that involves human historic responsibility and subsequent resource limits faced by animals. These may provide a reason — though not necessarily an over-riding one — to support interventions that relieve extreme suffering and that, in doing so, do not significantly compromise agency. In some cases, protection or promotion of other values such as biodiversity, ecosystem flourishing, and wildness will also be important in informing decision-making, alongside scientific, aesthetic and cultural values (Palmer *et al.* 2023). The complexity of such decisions mean that decision-makers will need to engage stakeholders in some form of dialogue, to ensure deliberation about what the goals are for the ecosystems in question and for the animals — however they are categorised. Here, it is interesting to note that the current management of the Sable Island horses has, at least in part, been shaped by involvement of the Canadian public, while there has not been a similar involvement of the Scottish public in the case of the Soay sheep. Increasingly, climate change will add a further contextual dimension: as conditions shift, so too may the frequency and severity of seasonal starvation, and the human causal responsibility we have identified as historically distant becomes, in this respect, more immediate and ongoing.

## Data Availability

The only hitherto unpublished data that we present in the paper are those underlying Figures 1 and 2. We are happy to share the dataset for these two figures upon written request. Regarding Figure 1, we were given written permission by Philip McLoughin to present the data; and regarding Figure 2, we were given written permission by Dan Nussey to present the data in this paper.

## Long descriptions

### Table 1. Long description

This table divides animals into various groups according to different relationships with people. It distinguishes between animals that are domesticated or undomesticated in terms of selective breeding, and those that are dependent on humans (managed by humans) or independent of humans (lacking human care).

Navigate back to Table 1..

### Figure 1 Long description

This figure illustrates the fluctations in population size of Sable Island horses from 1961-2025. It shows that population can shift significantly, with significant drops in some years, and significant rises in others, but with overall an increase in population

Navigate back to Figure 1.

### Figure 2 Long description

This figure shows how there has been significant fluctuation in the population of Soay sheep on Hirta Island from 1985-2025. In some years the population more than halved. Over time however the table shows that there has been an increase in population.

Navigate back to Figure 2.
